# Liver Transplantation in Alcohol-Associated Hepatitis. Benefits and Limitations of Psychosocial Selection and Support in Alcohol Relapse. The Experience of a Tertiary Center in Italy

**DOI:** 10.3389/ti.2024.13451

**Published:** 2025-01-06

**Authors:** Luca S. Belli, Adelaide Panariello, Giovanni Perricone, Paola Prandoni, Raffaella Viganò, Chiara Mazzarelli, Chiara Becchetti, Sara Giacalone, Giovanna Donvito, Sara Conti, Paolo A. Cortesi, Elena Roselli, Gianpaola Monti, Marco Carbone, Luciano G. De Carlis, Mauro Percudani

**Affiliations:** ^1^ Hepatology and Gastroenterology Unit, Azienda Socio Sanitaria Territoriale Grande Ospedale Metropolitano Niguarda, Milan, Italy; ^2^ Mental Diseases Unit, Azienda Socio Sanitaria Territoriale Grande Ospedale Metropolitano Niguarda, Milan, Italy; ^3^ Unit for Continuity Hospital-Territory Care Azienda Socio Sanitaria Territoriale Grande Ospedale Metropolitano Niguarda, Milan, Italy; ^4^ Research Centre on Public Health (CESP), University of Milano-Bicocca, Monza, Italy; ^5^ Intensive Care Unit, Azienda Socio Sanitaria Territoriale Grande Ospedale Metropolitano Niguarda, Milan, Italy; ^6^ General Surgery and Transplantation Unit, Azienda Socio Sanitaria Territoriale Grande Ospedale Metropolitano Niguarda, Milan, Italy; ^7^ Department of Medicine and Surgery, University of Milano-Bicocca, Milan, Italy

**Keywords:** alcohol-associated hepatitis, liver transplantation, alcohol use disorder, mortality, alcohol relapse, psychiatric conditions

## Abstract

Patients with severe alcoholic hepatitis SAH may suffer of undiagnosed psychiatric illnesses, typically depression. Assessment of prevalence and potential impact of psychiatric disturbances on alcohol relapse after LT, were the main objectives of this study. One hundred consecutive patients with SAH from April 2016 to May 2023 were analyzed. All patients were evaluated by an integrated team including psychiatrists, addiction specialists and social workers. Thirty (30%) were listed, of whom 25 underwent early liver transplantation (eLT) after a median time of 36 days from the index episode of SAH with a median model for end stage liver disease (MELD) score of 36, whereas 33 (33%) were excluded, with psycho-social issues being the main cause of exclusion in 18 patients (54.5%). Twenty-four patients (96%) are currently alive after a median follow-up of 32 months from LT. Sixteen transplanted patients had major depression with or without anxiety, with 10 patients (33%) being treated with antidepressants post-LT. Overall, 4 patients (16%) relapsed into alcohol consumption after liver transplantation and 1 died of alcohol related liver disease (4%). From this experience emerged that psychiatric comorbidities are highly prevalent among patients with SAH and that their diagnosis/treatment contributed to mitigate the risk of alcohol relapse.

## Graphical Abstract

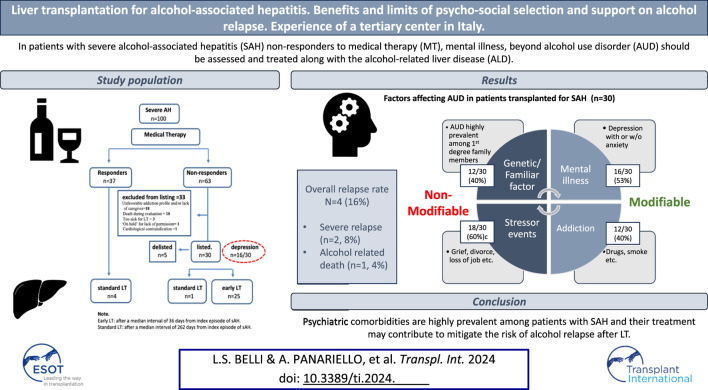

## Introduction

**FIGURE 1:**
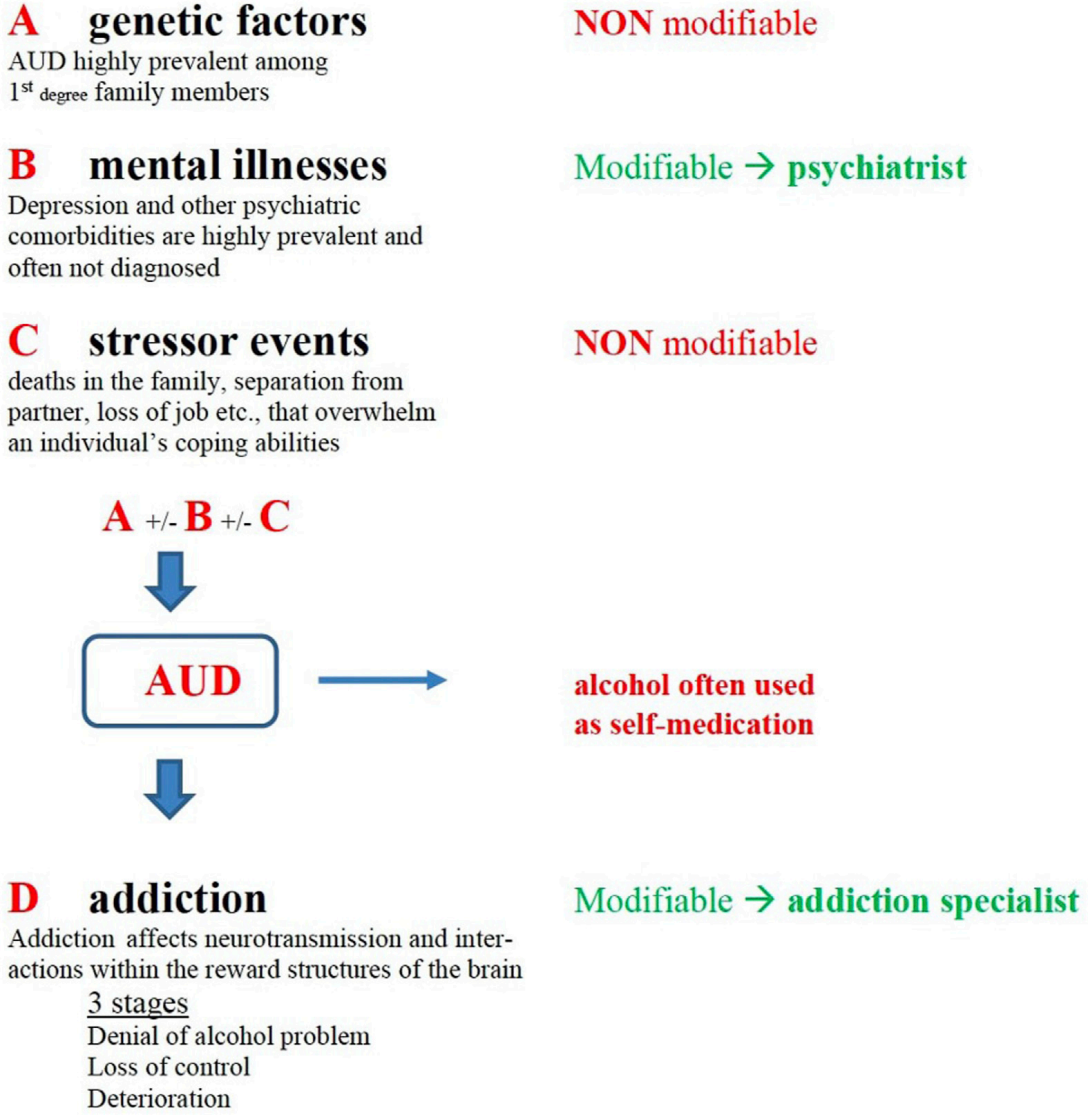
Modifiable and non-modifiable factors in patients with AUD Modifiable and non-modifiable factors in patients with AUD (adapted from the American Society of Addiction Medicine, Board of Directors, April 2, 2011).

Early liver transplantation (eLT) is considered the treatment option with the best outcomes in terms of survival for a select group of patients with severe alcohol-associated hepatitis (SAH) [1–7]. However, there are barriers to implementing programs due to scarcity of resources, ethical issues, and not least stigma [8]. A key issue is that many patients who develop SAH have unidentified or untreated psychiatric conditions that can range from depression to anxiety and personality disorders [9–12], conditions that hepatologists are not well-trained to treat and that may favor alcohol use disorder (AUD) [13–16]. As a matter of fact, a frequent interplay exists between psychiatric conditions (typically depression), genetic factors (familiarity for AUD), and stressful life events (i.e., deaths in the family, job loss, frustrating life events), with mental disorders (psychiatric illness and AUD) being potentially modifiable with medical treatments (MT) (Figure 1). Notably, undiagnosed depression is one of the mental health conditions in which environmental triggers can lead to alcohol abuse with alcohol being the self-medication that helps patients control their profound suffering and sadness. Identifying and implementing a parallel psycho-social pathway for patients before and after liver transplantation (LT) is imperative, to ensure not only the best candidate selection but also to minimize the risk of alcohol relapse after LT.

In this study we aimed to describe:

the psychosocial selection process used in a tertiary Center in Italy with a focus on the prevalence of psychiatric conditions and their response to treatment;
the reasons for not listing patients who do not respond to MT;
the benefits and limitations of psychosocial interventions on alcohol relapse after LT in our region.

## Patients and Methods

### Study Population

In total, 100 consecutive patients with clinically diagnosed SAH [17] admitted to our unit between April 2016 and May 2023 were included in this study and clinical and biochemical data were prospectively registered in a bespoke database. The severity of alcoholic hepatitis (AH) was assessed using Maddrey’s discriminant function (MDF) and the model for end-stage disease - sodium (MELD-Na) score.

### Data Collection

For each included patient the following parameters were collected: demographic characteristics (age at admission, sex, ethnicity), severity of liver disease (MDF, MELD-Na, Lille score), use of corticosteroids (CS), reasons for not using CS. Information regarding alcohol consumption and behavioral habits before LT, the amount of alcohol consumption in units/day, the duration of alcohol abuse, drug use, tobacco use, previous attempts at alcohol rehabilitation, legal issues, and family history of alcohol misuse were also recorded. In patients who underwent LT the following parameters were recorded: MELD-Na score at LT, explant histology, patient and graft survival, interval from admission to waiting list (WL) registration, interval from WL registration to LT, post-LT major complications, post-LT hospital stay, post-LT alcohol relapse, and interval from discharge to alcohol relapse.

Informed written consent was obtained from patients or their relatives for all participants. The study was approved by the Institutional Review Board and was conducted in accordance with both the Declaration of Helsinki and local law. Six of the 30 patient LT recipients were also included in a previous publication [6].

### Definitions

#### Response to Medical Treatment

Response to MT was based on the Lille model or a continuous reduction in MELD score reflecting a favorable trajectory of liver function.

#### Early vs. Standard LT

We adhered to the definition of the ACCELERATE study [3] which defines the window for eLT related to AH within 6 months of the index episode of SAH.

#### Post-LT Alcohol Relapse

Post-LT alcohol relapse was defined as any type of alcohol intake based on patient and family member interviews. Mild relapse: occasional slip, less than 1 per month. Moderate relapse: continuous drinking at daily doses within the recommended standards of up to 4 drinks/day for men and 3 drinks/day for women. Severe relapse: regular use above the recommended standards or with associated morbidity or mortality [18–20].

#### Risk Factors for Relapse to Alcohol Use

The following risk factors were collected: psychiatric co-morbidities, history of polysubstance abuse, drinking from a young age, family history of AUD, sub-optimal social support, failed attempts at alcohol rehabilitation, and smoking in the 6 months before transplant. A careful assessment of risk factors was used to optimize care before and after LT. All patients were recommended to join the local service for addiction surveillance and behavioral therapy. Since January 2023, patients with more than 2 risk factors were followed up by mental health specialists at the LT center.

### Exclusion Criteria for LT

Patients were considered not eligible for liver tranplant if one or more of the following conditions were present 1) poor awareness of AUD and lack of willingness to abstain; 2) unsatisfactory Global Assessment of Functioning or GAF, (lower than 70; see details in the Psychiatric and psychologic evaluation sub-chapter); 3) psychiatric disturbances that are not deemed treatable; 4) presence of significant cognitive impairment as assessed by neuro-psychologic tests prescribed by a dedicated psychiatrist on clinical suspicion; 5) inadequate social support and housing conditions 6) ongoing substance use disorder other than cannabis and methadone. The presence of at least 1 of the aforementioned conditions identified a patient with an unfavorable addiction/social profile.

In contrast with what was established in the study by Mathurin [1], patients with undiagnosed psychiatric disorders deemed treatable and patients with prior liver decompensation in case they had never been evaluated and supported by a mental health professional in the past, were not upfront excluded.

#### Psychiatric and Psychological Evaluation of the Whole Cohort

A dedicated psychiatrist and a psychologist evaluated all LT candidates in conjunction with the patient’s caregiver, usually a family member, and a social assistant when needed. The evaluation focused on four major aspects:

○   Severity of AUD according to the criteria of the diagnostic and statistical manual of mental disorders (DSM-5) [21], which identifies 3 classes (mild, moderate, severe) depending on the number of symptoms. Insight into AUD, coping skills, awareness, and agreement to adhere to lifelong alcohol abstinence were also assessed.
○   Presence of potentially treatable psychiatric disorders (depression, anxiety, personality disorders). To this aim, a symptom checklist (SCL 90) [22] was administered as a first screening tool to all potential LT candidates who were able to complete it. The Hamilton depression rating scale (HAM-D) and the Hamilton anxiety rating scale (HAM-A) were used to stratify the severity of depression and anxiety and for monitoring.
○   Since suffering from AUD does not only negatively affect the health of the patients, but also their social, educational, and occupational functioning, all these domains were evaluated using the “global assessment of functioning” or GAF scale [23], which is commonly utilized by psychiatrists to rate the impact of mental disease on daily life. It is divided into 10 sections and measures the extent to which a person’s symptoms affect their day-to-day life on a scale of 0–100. The higher the score, the better the patient can handle daily activities.
○ Presence of socioeconomic deprivation, particularly unemployment, poor housing conditions and lack of caregivers [24, 25]. In selected cases, a neuropsychologist was involved to exclude cognitive impairment related to alcohol or other etiologies.

#### Post-Transplant Follow-Up and Relapse Prevention Interventions

Patients were followed up every week for the first month after LT, then every month until 3 months, and then at least 6, 9, 12, 18, 24, 30, 36, 42 and 48 months thereafter. All patients were recommended to abstain from alcohol after LT and were routinely interviewed to elicit any alcohol use. Specific toxicological tests such as ethyl-glucuronide (ETG) in urine and hair were performed in patients with suspected alcohol relapse. Indirect markers of possible alcohol abuse (mean corpuscular volume [MCV], aspartate transaminase [AST], alanine transaminase [ALT], γ-glutamyl transpeptidase [GGT]) were also evaluated. All patients were evaluated by a dedicated mental health specialist team at the transplant center (psychologists or psychiatrists) at least once a year while also being referred to the local service for addiction. Patients with major depressive disorder were maintained on a predetermined strict surveillance with the dedicated psychiatrist at the transplant center with additional visits that were adjusted as clinically required. If a patient lapsed or relapsed after LT, an intensive individualized program was also initiated at the transplant center.

### Statistical Analysis

A descriptive analysis of the cohort was carried out on the total population. Continuous and categorical variables were summarized by absolute and relative frequencies, and median and interquartile range (IQR), respectively. A further analysis focused on non-responders and among them it compared the characteristics of patients listed for early-LT and those not listed. Group characteristics were compared using the Wilcoxon signed-rank test, while categorical ones were compared using Fisher’s exact test. Time was measured from the first day of hospitalization to the last known date of follow-up or date of death from any cause. Survival analyses, using the Kaplan-Meier method, were carried out overall and stratified for the following groups: responders, non-responders listed, and non-listed non-responders. All statistical analyses were conducted using R V.4.3.1 (R Core Team, Vienna, Austria).

## Results

During the study period, 100 consecutive patients with SAH were admitted to our Unit; in total, 63 (63%) were men, the median [IQR] age was 51 [44–56] years, 95 (95%) were Caucasian, and 70 (70%) had been referred from other hospitals. A history of prior liver decompensation was present in 29 patients (29%). At admission, the median [IQR] MDF was 72.1 [52–101] and the median [IQR] MELD score was 25.9 [23–30] (Table 1). According to the National Institute on alcohol abuse and alcoholism (NIAAA) criteria, the diagnosis of AH was “probable” in 71 patients and “definite” in the 29 patients who underwent a liver biopsy. An underlying liver cirrhosis was present in 98 patients (98%).

**TABLE 1:** Baseline characteristics of 100 patients with severe alcohol-associated hepatitis.

Gender	
Male patients, n (%)	63 (63.0)
Age, median (IQR)	51.5 (44.0–56.5)
Ethnicity, n (%)	
Caucasian	95 (95.0)
Other	5 (5.0)
Referred from another hospital, n (%)	70 (70.0)
Previous episode of liver decompensation, n (%)	29 (29.0)
Maddrey’s Discriminant Function, median (IQR)	72.1 (51.9–100.9)
MELD score, median (IQR)	25.9 (22.8–30.1)
MELD-Na score, median (IQR)	28.5 (25.0–32.3)
AH diagnosed as “probable”	71 (71.0)
AH diagnosed as “definite” (histologic confirmation)	29 (29.0)
Underlying cirrhosis, n (%)	98 (98.0)
Confirmed histologically at index episode of AH	30 (30.0)
Psychosocial characteristics	
Alcohol consumption, units/day, median (IQR)	10 (9.0–15.0)
Duration of alcohol consumption, years, median (IQR)	20 (15.0–30.0)
Active smokers, n (%)	45 (45.0)
Illicit substance users, n (%)	10 (10.0)
Living with partner, n (%)	60 (60.0)
AUD in first grade family members, n (%)	40 (40.0)
Previous detoxification attempts, n (%)	30 (30.0)
Psychiatric comorbidities, n (%)	55 (55.0)
Depression	22 (22.0)
Depression and anxiety	13 (13.0)
Depression and personality disorder	6 (6.0)
Anxiety	7 (7.0)
Personality disorder	7 (7.0)

Abbreviations: ACLF, acute on chronic liver failure; GAF, global assessment of functioning; MELD, model for end-stage liver disease; MELD-Na, model for end-stage liver disease-sodium; sAH, severe alcohol-associated hepatitis.

The median [IQR] alcohol intake was 10 [9–15] units/day, with a median [IQR] duration of alcohol intake of 20 [15–30] years. A total of 45 patients (45%) were active smokers and 10 (10%) reported the use of illicit drugs. A total of 40 patients (40%) had a first-degree family member suffering from AUD. In total, 30 (30%) patients failed previous attempts of detoxification. All patients met the criteria for severe AUD according to DSM-5 [21] and 55 patients (55%) met the criteria for a mood disorder and/or anxiety disorder (Table 1). Depression was the most common mental disorder, observed in 41 (41%) patients in the cohort and categorized as moderate-severe in 34 (34%) cases.

### Outcome After Response or No Response to Medical Treatment

**FIGURE 2:**
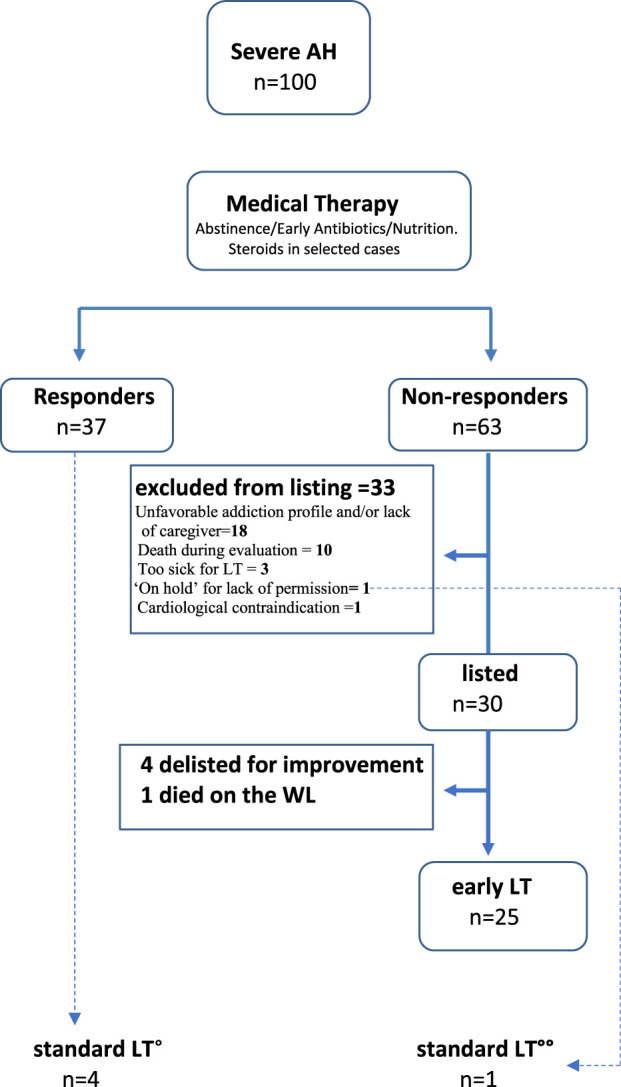
Disposition of patients. Note: Early LT, after a median interval of 36 days from index episode of sAH. Standard LT, after a median interval of 262 days from index episode of sAH. °4 patients who were discharged from hospital re-decompensated again for reasons not related to alcohol relapse and were listed for standard LT. °°One “irregular immigrant” could be listed only when regularized 7 months after index episode of sAH.

The main components of MT were alcohol abstinence, early identification and appropriate treatment of infections, nutrition, and the use of CS in selected patients (Figure 2). The main reasons for not using CS were spontaneous decrease of bilirubin (25 patients, 25%), confirmed or presumed infection (16 patients, 16%), acute on chronic liver failure (ACLF) grade 3 (17 patients, 17%), rapid worsening (9 patients, 9%), MELD score >30 (7 patients, 7%).

In total, 63 (63%) patients were non-responders to MT (Figure 2). Of these, 30 (30/63, 47%) were listed and 25 (25/63, 39.7%) underwent eLT after a median interval of 36 days after the index episode of SAH with a median MELD score of 36, and 33 patients were excluded from were considered not suitable for a LT for the following reasons: unfavorable addiction profile (lack of awareness of AUD) with or without an adequate caregiver (18 patients); death during the evaluation process [10 patients: 8 due to sepsis, 1 due to acute distress respiratory syndrome (ARDS) and 1 due to liver failure]; too sick for LT (3 patients) severe aortic valve regurgitation (1 patient); and one patient was ‘on hold’ while waiting for his residency permit (Figure 2). This last patient received a standard LT 7 months after the index episode of SAH.

A total of 37 patients responded to medical treatment but four of them decompensated again in the following months for reasons not related to alcohol abuse and received standard LT more than 6 months after the index episode of SAH, after a median of 236 days (Figure 2).

When comparing the psycho-social characteristics of patients listed for eLT with those excluded, notably an unfavorable addiction profile and socio-economic deprivation including lack of an adequate caregiver were the main reasons for exclusion in 58% of the cases (Table 2).

**TABLE 2:** Psychosocial characteristics of patients listed for early LT compared to those excluded from placed on the waiting list for transplant.

	Listed for LT n = 30	Excluded from early LT n = 33	Total n = 63	*P*-value
Gender				
Male patients, n (%)	21 (70.0)	19 (57.6)	40 (63.5)	0.447
Age, median (IQR)	51.5 (46.3, 56.8)	53.0 (45.0, 58.0)	52.0 (45.5, 57.5)	0.757
Ethnicity, n (%)				0.675
Caucasian	29 (96.7)	30 (90.9)	59 (93.7)	
Other	1 (3.3)	3 (9.1)	4 (6.3)	
Referred from other hospitals, n (%)	27 (90.0)	25 (75.8)	52 (82.5)	0.248
Previous episode of liver decompensation, n (%)	8 (26.7)	11 (33.3)	19 (30.2)	0.763
Psychosocial characteristics				
Alcohol consumption, units/day, median (IQR)	10.0 (7.25, 15.0)	10.0 (8.75, 15.5)	10.0 (8.0, 15.0)	0.599
Duration of alcohol consumption, years, median (IQR)	22.50 (20.0, 30.0)	20.00 (6.5, 30.0)	20.00 (19.3, 30.0)	0.442
Active smokers, n (%)	10 (33.3)	17 (51.5)	27 (42.9)	0.228
Illicit substance users, n (%)	2 (6.7)	3 (9.1)	5 (7.9)	1.000
Living conditions, n (%)				0.381
Living with partner	23 (76.7)	18 (54.5)	41 (65.1)	
Living alone	4 (13.3)	7 (21.2)	11 (17.5)	
Living with parents	3 (10.0)	5 (15.2)	8 (12.7)	
Working conditions, n (%)				0.680
Actively working	14 (46.7)	12 (36.4)	26 (41.3)	
Retired	3 (10.0)	4 (12.1)	7 (11.1)	
Unemployed	13 (43.3)	15 (45.5)	28 (44.4)	
AUD in first grade family members, n (%)	12 (40.0)	11 (33.3)	23 (36.5)	1.000
Previous detoxification attempts, n (%)	9 (30.0)	9 (27.3)	18 (28.6)	1.000
Psychosocial characteristics leading to exclusion from listing^a^	0			
Poor awareness of AUD	0	18 (54.5)		
Lack of caregiver	0	9 (27.2)		
GAF<70	0	6 (18.1)		
Cognitive impairment	0	2 (6)		
Untreated psychiatric condition or active substance use		0		
Psychiatric comorbidities, n (%)				
Depression	8 (26.7)	6 (18.2)	14 (22.2)	0.659
Depression and anxiety	4 (13.3)	3 (9.1)	7 (11.1)	0.928
Depression and personality disorder	3 (10.0)	2 (6.1)	5 (7.9)	0.940
Anxiety	3 (10.0)	0 (0.0)	3 (4.8)	0.214
Personality disorder	0 (0.0)	4 (12.1)	4 (6.3)	0.138
MELD score at LT, median (IQR)*	36.0 (30–39)	—	—	—

Abbreviations. IQR: interquartile range; AUD: alcohol use disorder; GAF: global assessment of functioning; MELD, model for end-stage liver disease; sAH, severe alcohol-associated hepatitis. *calculated for the 25 patients who underwent e-LT, after a median interval of 36 days (23–69) after the index episode of SAH.

^a^ All 18 patients excluded for psychosocial issues, had poor awareness of their AUD; all other psychosocial characteristics are to be considered in addition to poor awareness.

### Psychiatric and Psychological Characteristics of Transplanted Patients

All transplant patients had a GAF higher than 70 points with a median of 80, reflecting some mild symptoms, or some difficulty in social, occupational, or school functioning, but generally functioning well. Depression (with or without concomitant anxiety or personality disorder) was diagnosed in 16 patients and was moderate or severe (according to the HAM-D scale) in 12 patients. Antidepressant medication was administered to 10 patients and 9 showed significant clinical improvement when reassessed 6, 12 and 24 months later (Table 3, Panel A). Anxiety alone was diagnosed in 5 additional patients and was treated with psychological therapy (Table 3, Panel B). Patients with depression and anxiety were regularly followed up in the psychiatric clinic at the transplant site.

**TABLE 3:** Number of cases with depression and anxiety disorders in the 30 patients who underwent LT stratified by severity (according to HAM-D and HAM_A classification) and their outcome after 6, 12 and 24 months of treatment.

	At LT	After 6 months	After 12 months	After 24 months
Severe Depression, n	2	0	0	0
Moderate Depression, n	10	1	0	0
Mild depression, n	4	0	0	1
Total	16	1	0	0

Severe anxiety, n	0	0	0	0
Moderate anxiety, n	2	0	0	0
Mild anxiety, n	3	0	0	0
Total	5	0	0	0

### Psychiatric Disturbances: Treatment and Results

Of the 16 LT recipients suffering from depression, two had severe depression according to the Hamilton Rating Scale (>25) and were treated with escalating doses of escitalopram up to a maximum dosage of 10 mg/day with complete remission of symptoms after 1 month. In total, 10 patients had moderate depression (Hamilton Rating Scale between 18 and 25) and were treated with lower escalating doses of escitalopram up to a maximum dosage of 8 mg/day with remission of symptoms after 1 month. One patient autonomously discontinued antidepressant therapy and had a relapse 6 months after LT. All patients received psychotherapy interventions in case of addiction to psychopharmacotherapy. Patients with mild depression and anxiety disorder received only psychotherapy interventions with a good recovery. No cases of severe anxiety requiring selective serotonin reuptake inhibitors (SSRIs) were present in our cohort. The follow-up period was 24 months with regular checks of improvement on the Hamilton depression rating scale and the Hamilton anxiety rating scale (Table 3).

### Issues Regarding Adherence to Local Services for Addiction

Once transplanted, all patients were recommended to join the local service for addiction surveillance and behavioral therapies, but long-term adherence was sub-optimal as 10 patients (40%) refused to maintain contact with the Addiction Unit mainly because they did not like it or because they perceived no benefit. Notably, 2 patients in stable condition were discharged by the addiction specialists after a variable period of care, mainly due to the need to devote limited resources to participants with an active issue. These 10 patients were otherwise regularly seen by the mental health specialists at our center at least once a year on the occasion of the visit with the transplant hepatologist. None of them has had an alcohol relapse to date.

### Outcomes With and Without LT: Survival and Alcohol Relapse

A total of 30 patients (30%) were transplanted, 25 after a median of 36 days (IQR) after the index episode of sAH (early-LT) and 5 after a median of 236 days (IQR 60–117) (sLT). After a median (IQR) follow-up of 32.2 months (IQR 9.5–61.4), 4 patients (13%) resumed alcohol intake after 3, 6, 30 and 36 months after LT, respectively. Alcohol relapse was harmful in 2 cases and one patient died of end-stage ALD. The 4 patients with alcohol relapse underwent stricter surveillance at our center and two of the three living patients are now abstinent.

Of the eight patients who were transplanted despite previous episodes of liver de-compensation before LT, 5 had undiagnosed moderate or severe depression that was considered curable and all of them had very strong family support. Only 1 relapsed despite regularly attending the local addiction unit and despite being closely followed up by a psychiatrist for his depression. Unfortunately, a very negative stressor event, (abandonment by his wife), occurred 15 months after LT which the patient was not able to cope with. (see AUD story of patient 1).

**FIGURE 3:**
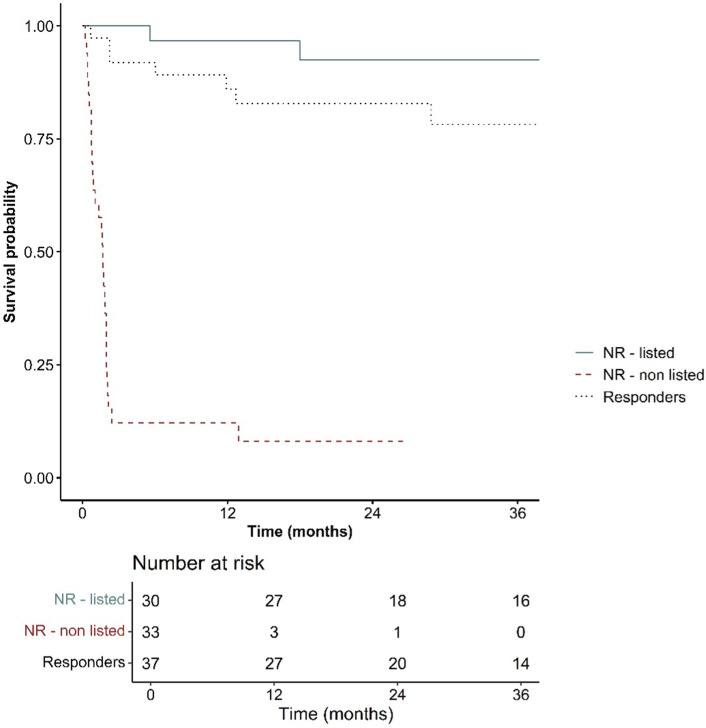
Survival probability.

The 24 month-survival of the patients excluded from transplant was 10% (Figure 3)

### Brief AUD Histories of the 4 Patients Who Relapsed

Patient n. 1. Before LT he had 2 admissions for AH, but was able to remain abstinent for 15 months between the 2 episodes and suffered from severe depression which was controlled with selective serotonin reuptake inhibitors (SSRIs), 8 mg escitalopram. His GAF was favorable, >70, he had a permanent job and his wife was a very motivated caregiver. After a thorough discussion within the team, he was accepted for LT on the condition that he follow an intensive individualized program of support from mental health specialists. Three months after the transplant while regularly attending the local addiction unit, he had a severe alcohol relapse which was triggered by the separation from his wife. Despite all of our efforts, he died 28 months later of alcohol-related end-stage liver disease. Patient n. 2. He started drinking 20 years before LT and 4 years before LT increased alcohol consumption to 2-3 bottles of wine per day after the death of his mother. His GAF profile was favorable (married, small child, actively working as a greengrocer, no psychiatric disorders). Unfortunately, he resumed occasional drinking 30 months after LT which became severe in the following months. His adherence to control visits has remained sub-optimal since then, but he is alive 62 months after LT with normal AST/ALT and slightly elevated GGT, and he keeps working as a “street” greengrocer. He is currently on 5 mg escitalopram, and 600 mg gabapentin. Patient n. 3. He resumed moderate alcohol consumption 6 months after LT, concurrent with a stressful event (separation from his wife and young child who had moved to Morocco). He was then started on an intensive support program with our mental health specialists and has remained abstinent since then. The family was reunited a few months later. At the last visit, he had normal LFTs and a stable job as a metalworker. No need for medication for AUD. Patient 4. Admitted for an index episode of alcohol-associated hepatitis in July 2018 while suffering from moderate depression for which he is currently being treated. Following the liver transplant, he returned to full active work and regularly attended the specialized addiction unit while being strongly supported by his very attentive wife. After 40 months he resumed occasional moderate alcohol consumption with some craving symptoms. He was started in an intensive program but still admits to occasional lapses. He is currently on 8 mg escitalopram and 800 mg gabapentin.

## Discussion

This study highlights the importance of integrated collaboration with a psycho-social team that includes dedicated psychiatrists, addiction specialists and social workers in order to identify patients suitable for LT and to implement therapies to help patients manage the risk of alcohol relapse after LT [26–31]. Thanks to this collaboration 96% of our cohort of patients with sAH being offered an LT are currently alive (Figure 3) and the alcohol relapse rate was limited (16%) after a median follow-up of 32 months.

Our selection process was different from that used in other Centers [1, 6, 30–33], particularly with respect to the following 2 clinical issues. First, central to the inclusion criteria in many centers was the requirement that the episode of sAH be the first decompensating event, on the assumption that a history of prior liver decompensation identifies patients who are less likely to remain abstinent after LT. We decided to adopt less stringent criteria since the majority of patients in the present cohort had never been referred to a mental health specialist for the presence of potentially treatable psychiatric disorders predisposing to AUD, nor had they been evaluated and supported by an addiction specialist. In the end, 8 patients with prior liver decompensating events were transplanted. Our experience follows and partly confirms that reported by Weinberg et al. [32], in whom 31 patients with prior decompensation were transplanted in the U.S. and were at significantly higher risk for any alcohol use after LT when compared with those without prior decompensation. Second, patients with moderate-severe depression, which is highly prevalent in patients with AUD, were not excluded.

Depression with or without concomitant anxiety or personality disorders was newly diagnosed in 41 patients (41/100, 41%) including 16 (16/30, 53%) LT candidates, who were effectively treated with antidepressants after LT and maintained on regular follow-up with the dedicated psychiatrist at the LT Center. Of the 4 classes of drugs currently available, namely, SSRIs (such as escitalopram, fluoxetine, paroxetine, sertraline, etc.), Serotonin-norepinephrine reuptake inhibitors (SNRIs, such as duloxetine, venlafaxine, etc.), Monoamine oxidase inhibitors (MAOIs such as isocarboxazid) and Tricyclic antidepressants (TCAs, such as amitriptyline, imipramine, etc.), only SSRIs were used in patients with advanced liver disease or LT recipients, due to safety issues. Even when using SSRIs, clinicians should be aware of possible drug interactions. Fluoxetine and paroxetine may cause a rise in tacrolimus and cyclosporine blood levels through inhibition of cytochrome P450 3A4 enzymes, while citalopram, escitalopram and sertraline have a limited effect on cytochrome P450 enzymes and were the first-line drugs whenever indicated. To our knowledge detailed data on psychiatric comorbidities have not been reported in this specific setting. This less stringent selection led to the applicability of eLT in 39% of non-responder patients, which is higher than what has been observed in other multicenter cohort studies where LT applicability was below 30% [1, 4, 6].

Regarding the main reasons for not listing patients who were non-responders to MT, an unfavorable addiction profile and socio-economic deprivation including lack of an adequate caregiver, accounted for the majority of the exclusions. We believe that an early referral to mental health specialists would be key to preventing a large percentage of patients with AUD from progressing to the more advanced stage of addiction. Unfortunately, early referral is not very common in our area. In the same vein, socio-economic deprivation including unemployment, poor housing conditions and lack of a caregiver are frequently exacerbated by AUD but are only marginally mitigated by current interventions of social assistance.

Alcohol relapse was documented in 4 patients, 13%, with 2 patients experiencing severe relapse and 1 patient dying from end-stage alcohol-related liver disease despite being closely followed up by our addiction specialist and psychiatrist. Notably a severe trigger event, typically a loss in the family, was present in 3 of the 4 patients who relapsed. Overall, only 60% of the patients were regularly followed up by a specialized addiction unit in the territory, as 40% either refused to maintain contact with local services or were discharged by local services after a variable period due to the limited resources allocated to addiction care in our area. This finding points to the limits of the use of addiction specialists outside the LT units, at least in the region of Lombardy. Based on this experience, we have decided that patients who are considered by experience with a higher risk of relapse, typically those with 2 or more risk factors, should be strictly linked to the integrated addiction specialists within our LT unit and that closer collaboration with addiction services in the territory needs to be implemented. Despite these drawbacks overall alcohol relapse and severe alcohol relapse were limited, at 16% and 8%, respectively, after a median follow-up of 32 months and we hypothesize that accurate diagnosis and control of depression were valuable tools in helping patients to maintain abstinence after LT.

We acknowledge some limitations of this study. First, the majority of patients were identified as having probable sAH with the diagnosis confirmed by histological findings in less than one-third of patients with an available pre-LT liver biopsy. We cannot exclude that some patients without a liver biopsy were misclassified although their clinical presentation was typical of AH. Second, biomarkers for the detection of alcohol use were not systematically used after LT which may underestimate alcohol relapse. This limitation was offset by lifelong post-transplant hepatology follow-up care at our transplant center, which consistently included inquiries about alcohol use, laboratory tests and ultrasound examinations for evidence of recurrent disease. Although rare or low-dose drinking was likely to be underreported, relapse with a negative impact on liver function would have been detected by the hepatology team. Third, the denominator in our study included only patients who were transferred to our Liver Unit as it was not possible to capture those patients who were referred to our Center but not transferred, as they were excluded by our mental health specialists after discussion with the mental health specialists of the local hospital or with the addiction specialist in the territory. Fourth, the median follow-up of 32 months after LT with a wide IQR does not allow a reliable assessment of alcohol relapse. In addition, as the vast majority of our patients were Caucasian, 95%, the conclusions of this study cannot be generalized to diverse populations. Finally, a control group of patients with untreated depression/anxiety was not available which limits the understanding of the extent to which diagnosis and treatment of depression/anxiety reduce relapse rates.

In conclusion, integrated collaboration with mental health specialists, psychiatrists, and addiction specialists may have been key to the initial success of the program although referral to addiction specialists outside the LT unit was suboptimal. We highlight the high prevalence of undiagnosed psychiatric comorbidities which are often curable, possibly contributing to mitigating the risk of alcohol relapse after liver transplantation.

## Data Availability

The raw data supporting the conclusions of this article will be made available by the authors, without undue reservation.

## Abbreviations

ACLF, acute on chronic liver failure; AD, acute decompensation; AH, alcoholic hepatitis; ALD, alcohol-associated liver disease; ALT, alanine transaminase; AST, aspartate transaminase; AUD, alcohol use disorder; CS, corticosteroids; DSM, diagnostic and statistical manual of mental disorders; ETG, ethyl-glucuronide; let, early liver transplantation; GAF, global assessment of functioning; GGT, γ-glutamyl transpeptidase; HAM-A, Hamilton anxiety rating scale; HAM-D, Hamilton depression rating scale; LT, liver transplantation; MDF, Maddrey’s discriminant function; MELD, model for end-stage liver disease; MELD-Na, model for end-stage disease - sodium; MT, medical treatment; NIAAA, National institute on alcohol abuse and alcoholism; sLT, standard liver transplantation; WL, waiting list.
